# Estimating point prevalence of COVID-19 in Qatar’s primary care registered population: an RT-PCR drive-through study protocol

**DOI:** 10.3399/BJGPO.2020.0160

**Published:** 2021-03-03

**Authors:** Mohamed Ahmed Syed, Ahmed Sameer Al Nuaimi, Hamda Abdulla A/Qotba, Hanan Al Mujjali, Mariam Ali Abdulmalik, Samya Ahmad Al Abdulla, Aisha Hussain Aladab, Kiran Govindan Kutty, Ehab Said Hamed

**Affiliations:** 1 Department of Clinical Affairs, Primary Health Care Corporation, Doha, Qatar; 2 Managing Director’s Office, Primary Health Care Corporation, Doha, Qatar; 3 Department of Clinical Operations, Primary Health Care Corporation, Doha, Qatar; 4 Pulmonology, Hamad Medical Corporation, Doha, Qatar

**Keywords:** COVID-19, epidemiology, primary health care, SARS-CoV-2

## Abstract

**Background:**

The first COVID-19 cases in Qatar were reported on 29 February 2020. As the epidemic progresses, essential epidemiological information is needed to facilitate monitoring of COVID-19 in the population and plan the pandemic response in Qatar.

**Aim:**

The primary aim of this cross-sectional study is to estimate the point prevalence of COVID-19 in Qatar’s primary care registered population.

**Design & setting:**

A cross-sectional study design will be utilised. One publicly funded health centre from each of three geographical regions in Qatar will be identified as a study location and set up to facilitate a drive-through for the study.

**Method:**

Primary Health Care Corporation (PHCC) is publicly funded and the largest primary care provider in Qatar. The study will include randomly selected individuals from the full list of PHCC's registered population on its electronic medical records system. The sample selection will be done using a proportional to size sampling technique stratified by age, sex, and nationality representative of the overall PHCC-registered population. Considering the total population registered in PHCC, a sample of 2080 is proposed. A questionnaire will be administered to collect sociodemographic information, and nasal and throat swab samples will be taken. Data will be analysed to report overall symptomatic and asymptomatic point prevalence of COVID-19.

**Conclusion:**

This study, with the help of a randomly selected representative sample from Qatar’s primary care registered population, will provide results that can be applied to the entire population. This study design will closely represent a real-world scenario of the outbreak and is likely to provide important data to guide COVID-19 pandemic planning and response in Qatar.

## How this fits in

Primary care is the cornerstone of any health system. It plays a crucial role in managing the COVID-19 pandemic. This study protocol highlights how primary care can contribute to generating robust epidemiological information of the SARS-COV-2 in a timely manner to support monitoring using minimal resources.

## Introduction

According to the World Health Organization's (WHO's) situation report, on 31 December 2019, Chinese national authorities reported an outbreak of pneumonia with unknown aetiology.^1^ On 12 January 2020, the National Health Commission in China associated the outbreak to a seafood market in Wuhan (China) and shared the genetic sequence of the novel causative agent, a novel coronavirus.^1^

Coronaviruses are enveloped, non-segmented, single-stranded, positive-sense ribonucleic acid (RNA) viruses named after their corona- or crown-like surface projections seen on electron microscopy that correspond to large surface spike proteins.^2^ Coronaviruses in the recent past have come to attention as pathogens of emerging respiratory disease outbreaks such as severe acute respiratory syndrome (SARS) in 2002–2003 and Middle East respiratory syndrome (MERS) in 2012–2014. The newly identified coronavirus with its epicentre in Wuhan was labelled severe acute respiratory coronavirus 2 (SARS-CoV-2), and is also known as 2019 novel coronavirus (2019-nCoV) and coronavirus disease 2019 (COVID-19).^3^

COVID-19 very quickly spread to other parts of China and the world. First imported cases were reported in Japan, Thailand, and Republic of Korea between the 13 January and 20 January.^1^ The first 1000 cases were infected within 48 days, which is a significant rate compared with SARS and MERS, which took 4 months and 2.5 years, respectively.^4^ With 18 countries affected and as the outbreak continued to spread globally, the WHO declared it a Public Health Emergency of International Concern on the 30 January 2020.^5^ With 118 000 cases in 114 countries, and 4291 deaths, the WHO declared the COVID-19 outbreak a pandemic on 11 March 2020.^6^

Primary care is the cornerstone of any health system. A strong primary care is often seen as a solution for the challenges that healthcare systems face.^7,8^ During pandemics, primary care is the frontline against emerging infectious diseases in communities. It provides infrastructure and plays a variety of key roles such as disease surveillance, diagnosis and treatment, prevention, patient education, and so on.^9^ During the peak week of a pandemic, it is estimated there could be a surge in primary care visits.^10^ These present challenges and opportunities in primary care.

The first COVID-19 cases in Qatar were reported on 29 February 2020. ^11^As SARS-CoV-2 continues to spread in the country, there is an urgent need to understand its epidemiology in primary care to plan resources and manage a response. Reverse transcriptase-polymerase chain reaction (RT-PCR) is routinely used to confirm diagnosis of COVID-19.^12^ While RT-PCR diagnostic testing is being conducted extensively across Qatar, similar to most other countries, it is undertaken to test individuals based on specific criteria (symptoms, profession, risk status, and so on) or close contacts of individuals who have tested positive^13–15^ on an ad hoc basis. In general, testing strategies of countries are passive.^16,17^ Furthermore, the majority of RT-PCR surveillance studies conducted to date include convenience samples, specific population groups and geographical regions, and so on.^18–20^ Such approaches do not provide an accurate picture of disease spread in the general population. More robust approaches are required to support key stakeholders and decisionmakers in order to implement tailored public health interventions.

The proposed study protocol is designed to provide a snapshot of the COVID-19 infections in Qatar’s primary care registered population. The study will be repeated based on passive surveillance findings and key stakeholders and decisionmakers' requirements. It will generate essential epidemiological information that will facilitate monitoring of COVID-19 in the population and plan the pandemic response in Qatar. The primary aim of this study is to estimate the point prevalence of COVID-19 in Qatar’s primary care registered population.

## Method

### Setting

Qatar is a peninsular Arab country with one of the highest gross domestic product (GDP) per capita in the world.^21^ It is known for extensive development over recent years and its ultra-modern lifestyle, attracting expatriates from all over the world. Qatar operates a universal, publicly funded healthcare system accessible to Qatari national and expatriates who hold a valid health card.^22^ A health card can be obtained at a cost of 100 Qatari riyals (approximately 28 US dollars).

Primary healthcare service in Qatar are delivered by the PHCC. The PHCC is the largest primary care provider in the country with 27 health centres (all accredited by Accreditation Canada International and distributed across three geographical regions: North, Central, and South) serving approximately 70% of the total population. All individuals who hold a valid health card are registered to a specific PHCC health centre.

### Study locations

One PHCC health centre from each of three geographical regions in Qatar will be identified as a study location. Each health centre will be set up to facilitate a drive through for the study.

### Sample selection

The study will include randomly selected individuals from a full list of eligible (aged ≥10 years) PHCC-registered population (*n* = 1 063 243, as of July 2020), which will be extracted from electronic medical records. The sample selection will be done using a proportional to size sampling technique stratified by age, sex, and, nationality representative of the overall PHCC-registered population (Table 1).

**Table 1.** Population sample selection

**Sociodemographic variable**	**Category**
Age group, years	≥60
	40–59
	18–39
	10–17
Sex	Female
	Male
Nationality	Non-Qatari
	Qatari

### Sample size calculation

Target fixed size strata will be used to achieve adequate representation of each strata. A total of 130 individuals will be randomly selected for each stratum. To adjust for non-response, 50% extra participants will be added (*n* = 65). The final strata sample size will be 195 and the resulting total sample size will be 3120 (Table 2).

**Table 2.** Sample size per population strata

	**Per strata**	**Total sample**
Targeted sample size	130	2080
Extra 50% to compensate for non-response rate	65	1040
Required sample size	195	3120

Weighting of each strata will be done at the analysis stage to represent the primary healthcare population. This approach is chosen to enable having a variable sample size in each strata after completing the survey without affecting the representativeness of the overall positivity rate estimate as population weights will be applied in the analysis stage.

The sample size formula, with an anticipated proportion of positive RT-PCR tests ranging between 0.5% and 5%, will be used. The targeted total sample size of 2080 is expected to estimate the PHCC population RT-PCR positivity rate (after weighting for population strata proportions) with 95% confidence and a margin of error (as a percentage of the expected estimate) ranging between 19% for the highest anticipated estimate of 5% and 60% error for the lowest anticipated estimate of 0.5% for positivity rate.

### Participant recruitment

Participants will also be sent an SMS invitation message 2 days in advance of a 2-day window during which the drive-through testing facilities at the three identified health centres will open. The SMS message will include information about the study and a link to a questionnaire survey to accept or decline the invitation. Participants will be invited to attend a study location in the same region as the health centre they are originally registered. A national campaign to publicise the study will also be initiated 2 days before its launch using television, newspapers, and social media to increase awareness and response rate.

### Data collection

Data collection at study locations will be undertaken as a drive-through. Participants will be seated in their cars and queue to be attended by a data collector. Data collection will be undertaken as a three-step process:

- Step 1: confirm participant is invited by SMS and verify details.
- Step 2: administer a questionnaire to collect information on sociodemographic factors (age, sex, nationality, residential address, education level, employment status, occupation, accommodation type, number of rooms and individuals living in household), lifestyle (physical activity, smoking status, fruit and vegetable consumption), history of chronic conditions, history of contact with a COVID-19 positive individual in the past 2 weeks, and presence of COVID-19 symptoms in the past 2 weeks (see Table 3).
- Step 3: collect a nasal and throat swab sample.

**Table 3.** COVID-19 symptoms in past 2 weeks

**Symptoms in past 2 weeks**
Fever 38 degrees Celsius or higher Chills Fatigue Muscle ache Sore throat Cough Runny nose Shortness of breath Wheezing Chest pain Other respiratory symptoms Headache Nausea or vomiting Abdominal pain Diarrhoea Loss of sense of smell Loss of sense of taste

Source: World Health Organization.^24^

### Laboratory test and notification of test results

The nasal and throat swabs collected will be analysed using RT-PCR. Participants with negative RT-PCR test results will be notified by SMS. Participants with positive RT-PCR test results will be contacted by the health centres where they are registered, and their health will be monitored and care provided as required.

### Analytical plan and anticipated results

All data will be collated at the end of study and subjected to quality assurance. For the purposes of the study, point prevalence will be defined as the number of active COVID-19 infections (identified by RT-PCR) over the total sample size and reported as proportions. All statistical analyses will be done using survey commands in SSPS (version 23).

The primary analysis will be undertaken to establish the overall point prevalence of COVID-19 in the PHCC primary care registered population. Secondary analysis will be undertaken to establish:

- point prevalence of COVID-19 by age, sex, residential area, nationality, educational level, occupation, contact with a suspected or confirmed COVID-19 case, and presence of COVID-19 symptoms in the PHCC-registered population.

- point prevalence by symptoms: COVID-19 cases from all COVID-19 cases in the PHCC-registered population.

The sensitivity and specificity of RT-PCR has been reported as 70% and 95%, respectively.^23^ These figures will be taken into account when reporting findings of the study.

The results of this study will provide information required to identify the extent of COVID-19 by sociodemographic factors, which will help identify social network and geographic areas with ongoing transmission. This information will help identify social networks and neighbourhoods, associated with increased levels of transmission that could benefit from targeted interventions.

## Discussion

RT-PCR is a diagnostic tool for COVID-19 and can contribute to understanding the spread of the disease in Qatar’s primary care registered population at a specific point in time. This study, with the help of a randomly selected representative sample, will provide results that can be applied to the entire population. This study design will closely represent a real-world scenario of the outbreak and is likely to provide important data to guide COVID-19 pandemic planning and response in Qatar.

The design of the study is its key strength. Identifying a representative sample of PHCC-registered population along with collection of sociodemographic data in combination with COVID-19 specific data will enable translation of findings to the overall PHCC-registered population. Furthermore, the design allows for rapid data collection, analysis, interpretation, and reporting of results to key stakeholders and decision makers. Once set up, it can be undertaken at required intervals to provide long-term, high quality data representative of the population.

There may be some limitations in this study. While efforts will be made to represent a real-world scenario, if the overall or strata response rate is low, it may not be possible to translate the study results. Also, while there is data collected, it will not be practical to collect significantly detailed information (such as behavioural and health status data) that may provide more in-depth insight into the outbreak, as the study is planned as a drive-through. Finally, positive RT-PCR diagnoses are being used as a proxy for COVID-19 incidence in the population. This may not accurately reflect incidence owing to limitations of the test itself (sensitivity, specificity, and so on).

This study will provide important information on the ongoing transmission of COVID-19 that represents a real-world scenario of the outbreak among the PHCC-registered population in Qatar. The study design will allow key stakeholders and decisionmakers to develop and implement tailored interventions for specific populations. In addition, it will provide important information on overall COVID-19 rates that will facilitate monitoring of COVID-19 in the population and plan the pandemic response in Qatar. The design of the study can be adapted for use across other healthcare settings in Qatar, as well as in other countries experiencing difficulties in understanding the epidemiology of the COVID-19 outbreak.
